# Effect of mouth rinses on bulk-fill composite microhardness - An *in vitro* study

**DOI:** 10.6026/973206300220300

**Published:** 2026-01-31

**Authors:** Pratheek V.S, Remya P. Mohanan

**Affiliations:** 1 Department of Conservative Dentistry and Endodontics, Government Dental College, Alappuzha, Kerala, India; 2 Department of Conservative Dentistry and Endodontics, Government Dental College, Kottayam, Kerala, India

**Keywords:** Bulk-fill composite, microhardness, mouth rinses, vickers hardness, dental materials, *in vitro*

## Abstract

The issue of loss of surface in bulk-fill composites through routine appurtenance to mouth rinses has not been adequately elucidated. Forty light cured bulk-fill composite specimen were made in this work using a standard Teflon mould and baseline Vickers microhardness measured. The samples were separated into 4 groups and dipped with 24 hours of artificial saliva, mouth rinse containing alcohol, desensitizing mouth rinse or alcohol-free mouth rinse. The use of one-way ANOVA and Tukey HSD test was used to analyze post-immersion microhardness. The tested mouth rinses decreased the surface microhardness of the composite, with alcohol content and low pH being found to be the leading factors that led to its deterioration.

## Background:

In modern dentistry, the resin-based composites have been used to form the direct aesthetic types of restorations which have mostly replaced dental amalgam because of patient's preference of tooth-colored restorations and the effects of mercury which has been linked to health hazards [1]. In spite of their high success, traditional composites allow clinical complications, most prominently polymerization contraction strain and the fact that incremental layering, as a meticulous and time- consuming method, is essential to secure the required depth of cure [2]. To overcome these shortcomings, there was the development of a new category of materials, which are referred to as bulk-fill resin composites. These materials are designed with improved light transmission, resin monomers with modified properties and the photo-initiator systems optimized in that they can be placed in 4-5 mm increments but with high conversion and low polymerization stress [3]. This protocol of placements simplifies making them very attractive due to the reduced chairside time. Although mechanical characteristics of bulk-fill composites are well-reported, their functionality and their survivability depend on their stability in the dynamic oral environment. Oral cavity is subjected to continuous exposure to chemical agents such as oral care mouth rinses that are given as a therapy to manage caries, gingivitis, or dentin hypersensitivity [4]. Active ingredients in these formulations may be chlorhexidine or fluoride, but also vehicles and solvents, the most commonly used of which is ethanol (alcohol). The role of alcohol is to dissolve active components and increase the penetration but it is also known to cause a negative effect on resin- based materials [5]. Alcohol may be used as a plasticizer, in that it enters the polymer framework, swelling and softening that framework, and may also drain off unreacted monomers and oligomers. This may deteriorate the mechanical characteristics of the composite such as surface hardness [6].

Various studies have been conducted on the influence of mouth rinses on dental restorations. A recent study by Ertürk-Avunduk *et al.* proved that both traditional and bulk-fill composites exhibited differential color changes after mouth rinse exposure, with alcohol and essential oil rinses causing greater discoloration in bulk-fill resins [7]. Mouth rinses with alcohol had the greatest effect on surface roughness of nanohybrid resin composite, according to Karademir *et al.* who also confirmed similar microhardness reduction in bulk-fill composites [8]. The study conducted by Dhingra and Kumari [9] stressed the damage that low-pH, alcohol-based mouth rinses can cause to bulk-fill resin composites when worn over a long period of time. Therefore, it is of interest to compare the Vickers surface microhardness of a commercially-available bulk-fill resin composite rinsed with three various mouthrinses containing alcohol, desensitizing mouthrinse and alcohol-free mouthrinse which was immersed in a number of test solutions and it was anticipated that the bulk-fill composite would not change significantly in microhardness.

## Materials and Methods:

## Study design and setting:

This experimental *in vitro* study was conducted at the Department of Conservative Dentistry and Endodontics, Government Dental College, Trivandrum.

## Specimen preparation:

A total of forty (N=40) cylindrical specimens of a commercially available bulk-fill restorative composite were fabricated. The composite material was spooned into a 3-millimeter-diameter-and 3-millimeter-high Teflon mould that was set on a polyester-lined glass slab. A little more material was added, a second polyester strip and a glass slide were placed on top, and then the material was gently compacted using hand pressure to remove any surplus and make the surface level and smooth. In accordance with the manufacturer's recommendations, each specimen was subjected to light curing for 40 seconds using an LED curing device that had an output intensity ranging from 450 to 500 mW/cm^2^. When the specimens were cured, they were taken out of the mould and placed in distilled water at 37°C for 24 hours until they were ready to be tested.

## Grouping and immersion protocol:

The forty specimens were randomly divided into four experimental groups (n=10 per group):

[1] Group A (Control): Specimens were immersed in artificial saliva (pH 7.2).

[2] Group B: Specimens were immersed in an alcohol-containing mouth rinse (Composition: Thymol 0.06% w/v, Eucalyptol 0.09%, Menthol 0.04%, Ethanol 21.6% v/v; pH 4.2).

[3] Group C: Specimens were immersed in a desensitizing mouth rinse (Composition: Aqua, Glycerin, Sorbitol, Potassium Nitrate, Sodium Fluoride; pH 6.7).

[4] Group D: Specimens were immersed in an alcohol-free mouth rinse (Composition: Camellia Sinensis Leaf Extract, Menthol, Cetylpyridinium Chloride, sorbitol pH 7.2).

Each specimen was individually stored in a sealed container with 20 mL of its respective solution at 37°C for a total of 24 hours to simulate short-term, intensive exposure. The pH of each mouth rinse was measured using a calibrated digital pH meter.

## Microhardness testing:

Vickers micro hardness tester was used to measure the surface microhardness. The specimens were evaluated twice: first before (T1) immersion and again after (T2) 24 hours in the solutions. The material was subjected to a 200-gram weight for 15 seconds for each measurement. The Vickers Hardness Number (VHN) was determined by making three indentations on the top surface of each specimen, with at least 1 mm of spacing between each indentation, and then averaging the diagonal lengths. At that moment, the specimen's microhardness was officially recorded as the mean of the three VHN readings.

## Statistical analysis:

SPSS (Version 18.0, SPSS Inc., Chicago, IL) was used to evaluate the data that we imported into an Excel spreadsheet. Each category has its own set of descriptive statistics computed, such as mean and standard deviation (SD). Baseline microhardness levels were found to be homogeneous. In order to compare the average microhardness values after immersion across the four groups, a one-way analysis of variance (ANOVA) was run. In order to find statistically significant differences between different groups, we used Tukey's HSD (Honestly Significant Difference) post-hoc test for pairwise comparisons. A p-value less than 0.05 were used to establish statistical significance.

## Results:

All 40 specimens were successfully fabricated and subjected to microhardness testing. The baseline microhardness measurements showed high consistency across all specimens, confirming the homogeneity of the samples before the immersion protocol was initiated Table 1. After 24 hours of immersion, all groups exhibited a decrease in mean microhardness. The descriptive statistics for post-immersion microhardness (T2) for all four groups are presented in Table 2. Group B (Alcohol-containing) showed the lowest mean VHN (44.50 ± 1.25), indicating the most significant softening effect. Group A (Control) demonstrated the highest mean VHN (53.60 ± 1.05), representing the least change. Groups C (Desensitizing) and D (Alcohol-free) had intermediate values. A statistically significant difference in the mean post-immersion microhardness values among the four groups was found by the one-way ANOVA test (F(3, 36) = 28.51, p < 0.001). These findings point to the fact that the surface hardness of the bulk-fill composite was significantly affected by the kind of immersion solution. Use of Tukey's HSD post-hoc test allowed for the identification of significant differences across subgroups. The findings from the comparisons made in pairs are reported in Table 3. Compared to the other groups, the alcohol-containing mouth rinse (Group B) stood out substantially (p < 0.001). Group A, the control group, had very high hardness compared to Group C, the desensitizing group, and Group D, the alcohol-free group. The desensitizing mouth rinse group and the alcohol-free mouth rinse group did not vary significantly (p > 0.05).

## Discussion:

Findings of this *in vitro* experiment showed that surface microhardness of a bulk-fill resin composite may be significantly lowered after a short-term exposure to commercial mouth rinses. The null hypothesis, which was that there would be no significant effect of mouth rinses on microhardness, was therefore rejected. The results show that the chemical structure of these oral hygiene products is significant in the degradation of surfaces of modern restorative materials. The greatest result was the considerable decrease in microhardness in the group which was placebo (Group B). A mean VHN of this group was about 20% less than the baseline value and much less than any other group. This is in line with a substantial amount of evidence indicating that ethanol is a strong solvent which may loosen the organic structure of resin composites [9, 10]. The process by which this degradation occurs is through the diffusion of alcohol molecules into the polymer network that enhances the liberation of the polymer chains- a process referred to as plasticization. The process decreases the intermolecular forces and causes swelling and lowers the mechanical properties like hardness and wear resistance [11]. More so, the ethanol is liable to leach unreacted monomers, oligomers and even some filler particles, further weakening the structural integrity of the material [12].

Group B mouthwash was not only not an alcohol-based preparation, but it also had a very low pH (4.2). Two of the ways in which an acidic environment could accelerate the degradation of resin composites include hydrolysis of the ester bonds in the dimethacrylate monomers (e.g., Bis-GMA, UDMA) and erosion of the filler-resin interface [13]. The enormous tenderness observed in this group must be attributed to the joint actions of low pH and high alcohol content, and these points out the potential clinical risk to patients who habitually take these products. The desensitizing (Group C) and alcohol-based (Group D) mouth rinses also resulted in statistically significant reduction in microhardness than the control group, but to a lower degree than the alcohol-based formulation. This implies that other factors may also cause changes in the surface as opposed to alcohol. These formulations are also composed of different organic compounds like glycerin, sorbitol and surfactants (e.g. poloxamer) which might have a small amount of plasticizing activity over time since it has the ability to diffuse into the resin matrix [14]. Even though the pH of these rinses was close to the neutral, their multifaceted chemical character is enough to cause the slightest changes to the surface. Interestingly, even the control group, which had been dipped in artificial saliva, experienced some slight (not statistically significant compared to Groups C and D) microhardness reduction at baseline. This has been explained by the fact that all dental resins have a phenomenon of water sorption. The polymeric filler material can be infiltrated by water molecules resulting in slight plasticization and hydrolytic degradation of the silane coupling agent in the filler-resin interface, resulting in a slight loss of mechanical properties [15].

These findings have implication in clinical practice. The hardness of the surface is an essential property that is directly proportional to the extreme resistance of the material to scratches and abrasive wear. He or she, a reduction of microhardness is indicative of the possibility that the composite restoration is more prone to degradation due to tooth brushing and masticatory forces that can result in loss of anatomical form, surface staining, and poor clinical survival [16]. These possible interactions should be noted by clinicians and they can also recommend alcohol-free, neutral pH mouth rinses to patients with large volumes of fillings in place to maintain the integrity of place restorations. This research has various shortcomings. Being an *in vitro* study, it is not able to completely recapitulate the complexity of the oral environment, which contains dynamic processes, which are salivary pellicle formation, enzyme activity, temperature, and mechanical loading. The 24-hour immersion is an aggressive and persistent challenge that is not intermittent and brief as used clinically. Future research needs to consider the outcomes of long-term and cyclic immersion regimes and the other material characteristics, including wear resistance, color stability, and surface roughness, to give a better representation of these material interactions.

## Conclusion:

Commercial mouth rinses that were tested significantly decreased the surface microhardness of the bulk-fill composite material after immersion with all of the commercial rinses. The highest negative effect that resulted in the heavy loss of microhardness was caused by the alcohol based mouth rinse that had least pH. Although they were not as harmful as the alcohol based formulation, desensitizing as well as alcohol free mouth rinses reduced microhardness significantly as compared to the control.

## Figures and Tables

**Table 1:** Baseline Vickers Microhardness (VHN) of All Specimens (N=40) Before Immersion

**Parameter**	**Mean VHN**	**Standard Deviation**
Baseline Hardness (T1)	55.54	0.23

**Table 2:** Post-Immersion Vickers Microhardness (VHN) Values (Mean ± SD) for Each Group (n=10)

**Group**	**Immersion Solution**	**pH**	**Mean VHN**	**Standard Deviation**
A	Artificial Saliva (Control)	7.2	53.6	1.05
B	Alcohol-containing Mouth rinse	4.2	44.5	1.25
C	Desensitizing Mouth rinse	6.7	50.2	3.2
D	Alcohol-free Mouth rinse	7.2	51.8	1.54

**Table 3:** Pairwise comparison of mean post-immersion microhardness using Tukey's HSD Test

**Comparison (Group I vs. Group J)**	**Mean Difference (I-J)**	**Std. Error**	**p-value**	**Significance**
A (Control) vs. B (Alcohol)	9.1	1.12	<0.001	Significant
A (Control) vs. C (Desensitizing)	3.4	1.12	0.021	Significant
A (Control) vs. D (Alcohol-free)	1.8	1.12	0.37	Not Significant
B (Alcohol) vs. C (Desensitizing)	-5.7	1.12	<0.001	Significant
B (Alcohol) vs. D (Alcohol-free)	-7.3	1.12	<0.001	Significant
C (Desensitizing) vs. D (Alcohol-free)	-1.6	1.12	0.485	Not Significant
*p-value < 0.05 indicates a statistically significant difference				
